# Z Probe, An Efficient Tool for Characterizing Long Non-Coding RNA in FFPE Tissues

**DOI:** 10.3390/ncrna4030020

**Published:** 2018-09-05

**Authors:** Manish K. Tripathi, Chidi Zacheaus, Kyle Doxtater, Fatemeh Keramatnia, Cuilan Gao, Murali M. Yallapu, Meena Jaggi, Subhash C. Chauhan

**Affiliations:** 1 Department of Pharmaceutical Sciences, University of Tennessee Health Science Center, Memphis, TN 38163, USA; czacheau@uthsc.edu (C.Z.); kdoxtate@uthsc.edu (K.D.); fkeramat@uthsc.edu (F.K.); myallapu@uthsc.edu (M.M.Y.); mjaggi@uthsc.edu (M.J.); 2 Department of Mathematics, University of Tennessee at Chattanooga, Chattanooga, TN 37403, USA; cuilan-gao@utc.edu

**Keywords:** long noncoding RNA, MALAT1, UCA1, NRON, Z probe, colorectal cancer, pancreatic cancer, breast cancer

## Abstract

Formalin-fixed paraffin embedded (FFPE) tissues are a valuable resource for biomarker discovery in order to understand the etiology of different cancers and many other diseases. Proteins are the biomarkers of interest with respect to FFPE tissues as RNA degradation is the major challenge in these tissue samples. Recently, non-protein coding transcripts, long non-coding RNAs (lncRNAs), have gained significant attention due to their important biological actions and potential involvement in cancer. RNA sequencing (RNA-seq) or quantitative reverse transcription-polymerase chain reaction (qRT-PCR) are the only validated methods to evaluate and study lncRNA expression and neither of them provides visual representation as immunohistochemistry (IHC) provides for proteins. We have standardized and are reporting a sensitive Z probe based in situ hybridization method to visually identify and quantify lncRNA in FFPE tissues. This assay is highly sensitive and identifies transcripts visible within different cell types and tumors. We have detected a scarcely expressed tumor suppressor lncRNA NRON (non-coding repressor of nuclear factor of activated T-cells (NFAT)), a moderately expressed oncogenic lncRNA UCA1 (urothelial cancer associated 1), and a highly studied and expressed lncRNA MALAT1 (metastasis associated lung adenocarcinoma transcript 1) in different cancers. High MALAT1 staining was found in colorectal, breast and pancreatic cancer. Additionally, we have observed an increase in MALAT1 expression in different stages of colorectal cancer.

## 1. Introduction

Long non-coding RNA (lncRNA) is a class of non-coding RNA that has gained significant traction with exponential publications and interest from the scientific community. A class of non-coding RNA, microRNA (miRNA), has been extensively studied in the last decade since its discovery. lncRNA on the other hand, provides a novel way of regulating gene expression and function at all levels of DNA, RNA or protein, however, its mechanism is still not clear; warranting new methods to elucidate their functions. In 2005 when 512 known lncRNA were systematically studied [1], it was not anticipated that this number would grow exponentially to 19,175 potentially functional lncRNAs in the human genome. Many of these potential lncRNAs identified by FANTOM5 (Functional Annotation of the Mouse/Mammalian Genome) analysis of cap analysis of gene expression (CAGE) data and overlapping expression qualitative trait loci (eQTL) have not been functionally described. It is anticipated that this number of lncRNAs will increase significantly [2]. Many of the lncRNAs are being characterized by revisiting the array datasets on publicly available resources [3,4,5,6,7]. LncRNAs, which are now at the center of various physiological and pathological processes, have been linked to various cellular pathways including the progression of different cancers and various diseases. Given lncRNA involvement in many diseases, it is imperative to invest efforts in understanding the roles of these new classes of regulators and further elucidate the mechanism.

Immunohistochemistry (IHC) for decades has been the primary diagnostic method of choice for identifying important biomarkers at the protein level in cancer and other diseases. The ability of IHC to detect important intracellular and extracellular proteins and receptors makes it an attractive diagnostic method in both laboratory and clinical settings. For example, the information gained by IHC in determining the status of breast cancer biopsies and helps in designing better therapeutic treatment interventions; a method applicable to other cancer diseases [8]. On the other hand, RNA in situ hybridization has been used widely to analyze messenger RNA (mRNA) in many studies [9,10,11], all of which lack visual representation. Quantitative reverse transcription-polymerase chain reaction (qRT-PCR), RNA sequencing (RNA-seq) and microarrays are the current gold standard for detecting lncRNA in cell lines and tissues. The problem, however, is the inability to differentiate cell types and populations within cancer tissues, as well as the compartmental location of the transcript. Any insight into the location and expression pattern of lncRNA may be valuable in developing better therapeutic treatments [12]. Most lncRNA studies have been based on bioinformatic analysis and correlation studies from publicly available databases and resources [2,6,7]. A major problem in understanding lncRNA’s role in cancer progression and other diseases is a lack of an efficient tool to characterize them. A new RNAScope technology was reported for the first time by Wang et al. [13] and was later used to investigate the level of mRNA expression for program death ligand-1 and its receptor PD1 in tissue cohorts of non-small cell lung cancer by Velcheti et al. [14]. If used as a corroborative diagnostic tool, this method may provide more confidence as a validation step to identify mRNA transcripts of the same protein biomarkers observed with IHC, or as an additional lncRNA detection method.

So far, there is currently no standard chromogenic methodology developed and reported for lncRNA detection in formalin-fixed paraffin embedded (FFPE) tissues. We are showing here that this technology is applicable to lncRNA visualization in FFPE tissues in a very user-friendly manner, just like the IHC procedure. We further envisage this technology to be used in diagnostic and clinical settings with our easy protocol. In the interest of having a specific, sensitive and reproducible lncRNA assay, we have standardized Z probe based on a chromogenic method in multiple cancer types. This assay can be used as a diagnostic detection assay for lncRNAs as cancer biomarkers. This method with rigorous steps, sensitivity and specificity, can improve patient lives through identification of localized gene expression within individual cancer cells as well as expression changes in different cell populations of cancer tissues.

## 2. Results

### 2.1. Schematic and Controls

**Figure 1:**
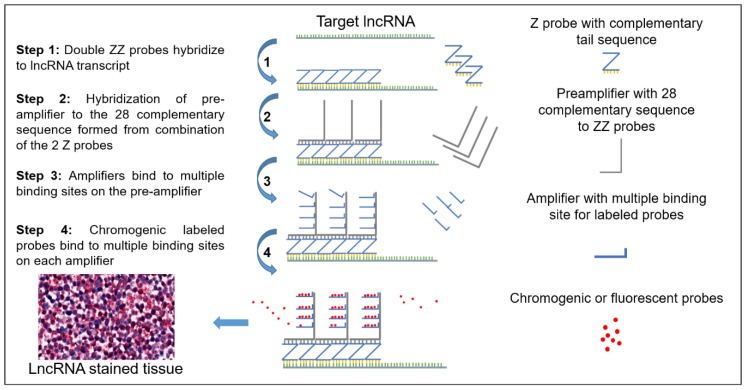
A schematic representation of the Z probe based RNAScope assay for long non-coding RNA (lncRNA) analysis. Starting with Z probes hybridizing with the target sequence creating double ZZs with up to 20 groups side by side. The pre-amplifier then binds to the complementary sequence on the 28-base tail (top of the ZZ). Pre-amplifiers contain multiple binding sites for amplifiers to bind to and the amplifiers also have multiple binding sites for labeled probes to bind. Upon chromogenic stain, the labeled probes fluoresce with a red color.

The schematic workflow of the RNAScope assay using Z probes is represented in Figure 1. Each Z probe has an 18–25 base complementary sequence to the target lncRNA and a 14 base sequence complementary to the pre-amplifier. Although three double ZZ probes are sufficient for a signal, Advanced Cell Diagnostics (ACD) designed all their probes to utilize 20 double ZZ probes, which covers around 1kb of the target transcript. Extending the number of double ZZ probes to 20 makes the assay robust and specific to target lncRNA according to ACD. With assay design and probe requirements, the chance of 20 double ZZ probes complimenting to a nonspecific site is extremely limited.

**Figure 2:**
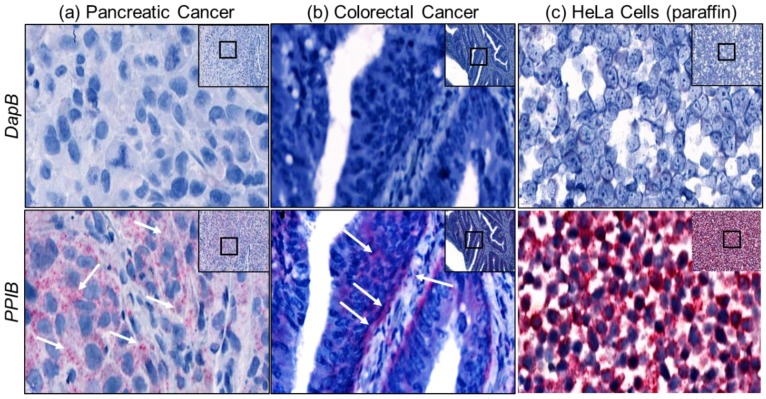
Validation and optimization of the Z-probe staining. Paraffin embedded, and sectioned, pancreatic cancer tissue, colorectal cancer tissue and HeLa cell pellet were stained with a negative control probe *DapB* (full form) and a positive control probe *PPIB* (full form). The *PPIB* stained well with pancreatic, colorectal and HeLa cells (bottom panel (**a**–**c**). 20× (inset) and 80× magnification using CaseViewer 2.2 software (3DHistech Ltd., Budapest, Hungary) scanned and analyzed on Pannoramic 250 Flash III (3DHistech Ltd.). Arrows point at specific staining.

Figure 2a–c shows the validation of the assay on FFPE sectioned pancreatic cancer, colorectal cancer and HeLa cells. *PPIB* (peptidylprolyl isomerase B), a human gene, and *DapB* (dihydrodipicolinate reductase), a bacterial (*Escherichia coli*) gene, were used as a positive control and a negative control probe respectively. *DapB* did not show any signal in pancreatic and colorectal cancer tissues, but *PPIB* stained nicely on the tissues (pancreatic and colorectal cancer, Figure 2a,b, respectively) as well as on the FFPE sectioned HeLa cells (Figure 2c). This result demonstrated that FFPE tissues can be trialed with the experimental probe staining with similar results as with the embedded cells.

### 2.2. Assay Validation

**Figure 3:**
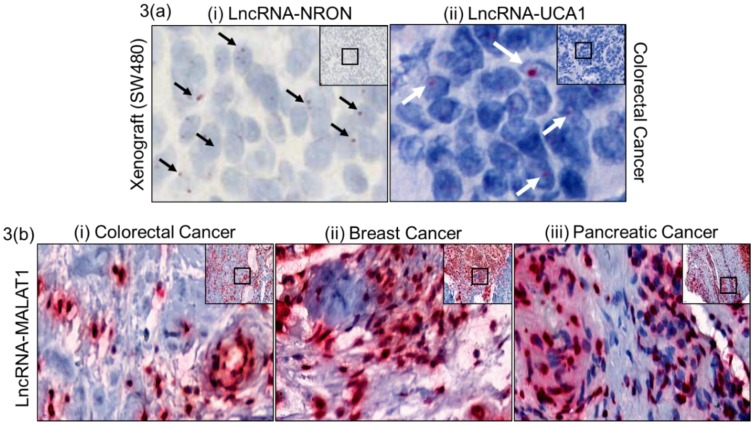
Paraffin embedded different human cancer tissues that have been Z probe stained for different lncRNAs. (**a**) Tumor suppressor lncRNA NRON (very low expression, non-coding repressor of NFAT) (**i**) and oncogenic lncRNA UCA1 (moderately expressed, urothelial cancer associated 1) (**ii**) stained in colorectal cancer tissue. (**b**) LncRNA MALAT1 (metastasis associated lung adenocarcinoma transcript 1) stained using specific Z-probes in paraffin embedded (**i**) colorectal cancer, (**ii**) breast cancer and (**iii**) pancreatic cancer tissues. 20× (inset) and 80× magnification using CaseViewer 2.2 software, scanned and analyzed on Pannoramic 250 Flash III. Arrows point at specific lncRNA signals.

In order to validate the assay method for identifying lncRNAs on FFPE tissues, we selected a low, moderately and highly expressed lncRNA. LncRNA NRON (non-coding repressor of nuclear factor of activated T-cells (NFAT)), is scarcely expressed and is very hard to quantify, even by qRT-PCR. Figure 3a(i) shows NRON signal in xenograft FFPE tissue. Similarly, the moderately expressed oncogenic lncRNA UCA1 (urothelial cancer associated 1) was very specifically located in colorectal cancer tissue in the epithelial population (Figure 3a(ii)), highlighting the sensitivity of the assay method. Further, we chose the highly expressed lncRNA MALAT1 (metastasis associated lung adenocarcinoma transcript 1) for staining in colorectal, breast and pancreatic cancer. MALAT1 showed a very prominent staining in these three cancer tissues, mostly in epithelial cell populations.

### 2.3. Quantitative Measure of Progression and Invasiveness

**Figure 4:**
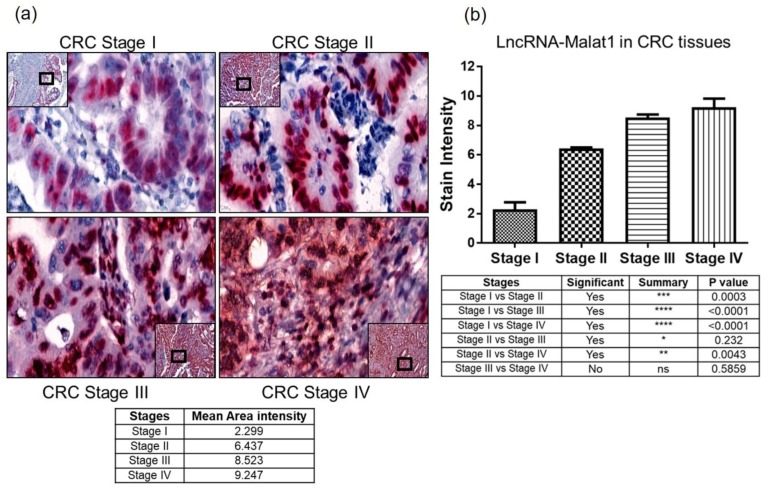
LncRNA-MALAT in different stages of colorectal cancer (CRC). Paraffin embedded different stages of colorectal cancer tissues were stained for lncRNA MALAT1 and quantitated for staining intensity. 60 CRC tissues, Stage I = 15; Stage II = 16; Stage III = 20; Stage IV = 9, were stained for lncRNA-MALAT1 (**a**) Stages I–IV CRC tissues show a differential stain for lncRNA MALAT1. Stain intensity correlates with the progression. (**b**) Quantitation of the lncRNA MALAT1 staining intensity was performed using Image J software. 10× (inset) and 80× magnification using CaseViewer 2.2 software, scanned and analyzed on Pannoramic 250 Flash III. Statistical analysis: One-way ANOVA and Tukey’ multiple comparison tests compare the mean of each column with the mean of other columns.

Prominent lncRNA MALAT1 staining encouraged us to utilize this assay to analyze MALAT1 as a cancer progression and invasiveness marker. Interestingly, lncRNA MALAT1 stained very well with respect to the progression of colorectal cancer. Figure 4a represents MALAT1 staining in different stages of colorectal cancer (CRC) with a mean area intensity represented below in different stages. Figure 4b represents the quantitative stain intensity in different stages of CRC tissues. The MALAT1 stain intensity is significantly correlated among different stage (except stage III and stage IV) progressions. Additionally, we observe higher MALAT1 expression in epithelial cells than in stroma cells. This might indicate a preferential expression within cell types and perhaps different a regulatory mechanism.

**Figure 5:**
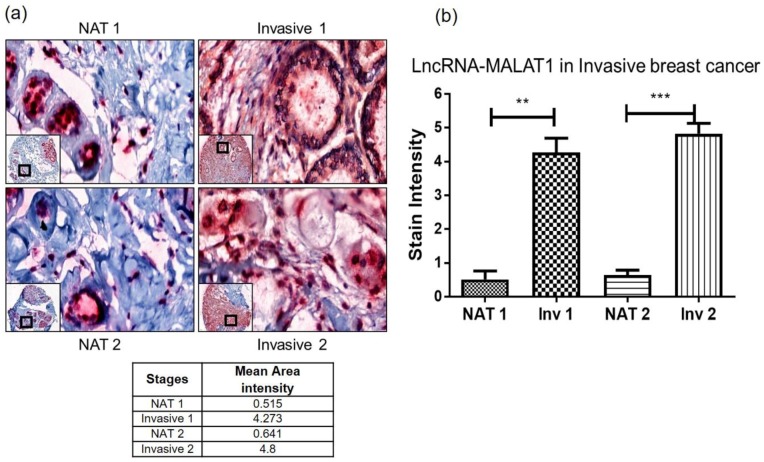
LncRNA MALAT1 expression in breast cancer. BioMax TMA BR243w containing 12 normal adjacent tumor (NAT) and 12 invasive breast carcinomas were stained and analyzed. (**a**) Matched breast cancer tissues (NAT vs. Invasive) from two different patients, 1 and 2, were stained for lncRNA MALAT1 using Z probe. The invasive breast cancer tissues have higher staining for lncRNA MALAT1 as compared to normal adjacent tumors. (**b**) Quantitation of the lncRNA MALAT1 staining intensity was performed using Image J software. 10× (inset) and 80× magnification using CaseViewer 2.2 software, scanned and analyzed on Pannoramic 250 Flash III. Statistical analysis: Unpaired *t*-test, ** *p* < 0.01, *** *p* < 0.001.

Finally, to verify whether this method can assess the invasiveness, we used a tumor microarray (TMA) with a normal to adjacent tumor (NAT) and matched invasive breast cancer tissues. Reassuringly, invasive breast cancer tissues have a higher MALAT1 staining (as shown in Figure 5a) and the mean area intensity is higher in both invasive tissues as compared to NAT. Figure 5b shows a significant difference in the staining intensity between NAT and invasive tissues indicating lncRNA MALAT1 as a marker of invasive breast cancer.

## 3. Discussion

Immunohistochemistry and qRT-PCR have long been the gold standard for investigating protein and RNA biomarkers respectively [8,15,16,17]. A lack of specificity, sensitivity, and reproducibility of IHC methods continue to limit our interpretation of data collected. This, in turn, makes clinical diagnosis challenging. While IHC is beneficial in identifying protein biomarkers, the assay is ineffective in detecting lncRNA in FFPE tissues, which is the limitation of the assay design. As our understanding of long non-coding RNAs and their role in cancer development and progression augments, a more specific, sensitive and reproducible tool is warranted. RNAScope allows for the amplification of the signal while maintaining tissue integrity, which is in contrast to real-time RT-PCR, as the tissues are homogenized during the process of extraction [13]. The strength of the Z probe assay lies on the fact that it utilizes approximately 1 kb of the target transcript and it employs 20 double ZZ binding probes side by side, which is definitely a more sensitive and robust assay than the conventional RNA in situ hybridization assay [9,10,11]. Use of the appropriate positive and negative controls strengthens the confidence in the results. The *PPIB* positive signal on xenograft HeLa cell and human cancer tissues simultaneously validated the procedure.

The tumor suppressor lncRNA NRON was one of the first lncRNAs identified from an unbiased screen [1]. NRON is a non-coding repressor of NFAT, and it is a tumor suppressor [18]. *NRON* is expressed in low amounts and its expression was difficult to visualize in human tumor FFPE tissue (data not shown), so SW480 (non-metastatic colorectal cancer cell line) xenograft FFPE tissue was used to detect NRON. Moderately expressed oncogenic lncRNA UCA1 has been studied for different pathways and interactions, resulting in cancer progression [19,20,21]. The assay in this study was able to show specific epithelial cell populations expressing UCA1 as a red dot. We were also able to show that MALAT1 is aberrantly expressed in TMAs of three cancer groups (colorectal, breast, and pancreatic), suggesting that lncRNA MALAT1 is associated with multiple cancers and its use as a therapeutic target should be investigated further. Though MALAT1 has been studied in different cancers as a poor prognostic [22,23,24,25], aggressive, and metastatic marker [22,26,27], most of the published data on MALAT1 lack visual representation of MALAT1 detection. All of the studies known so far have used RT-QPCR, RNA-seq or a curated database for their analysis. This is the first evidence of MALAT1 expression in FFPE patient tissues of breast, colorectal and pancreatic cancers by chromogenic staining, suggesting that lncRNA MALAT1 is associated with multiple cancers and its use as a therapeutic target should be investigated further. We have also shown progressive expression of MALAT1 across the different stages of colorectal cancer and were able to correlate the stages with the progression of CRC. To our confidence, the staining intensity of different region of interest (ROI) in the CRC tissues was statistically significant except for the Stage III and Stage IV comparison. More Stage III and Stage IV CRC tissues might help to increase the statistical significance. Invasive breast tissues have been shown to have higher MALAT1 expression [22,27,28,29], but for the first time this study gives visual evidence in multiple cancer types. Although we were only able to show UCA1 expression in CRC TMA, staining in TMA of other cancer groups will be investigated to determine if there is differential expression of UCA1 between cancer types.

As we continue to investigate the role of lncRNAs in cancer development and metastasis, this assay method will help provide a visual representation of this class of RNA molecules. Collectively, these data suggest that Z probe can be an efficient tool to characterize lncRNA molecules in FFPE tissues and cells. This Z probe-based detection method of lncRNA is sensitive and reproducible with high specificity. This assay alleviates all areas of concern by simultaneously detecting lncRNA molecules of interest while suppressing background noise with a novel Z probe pair design and signal amplifiers. In both laboratory and clinical settings, this assay can be utilized to corroborate the results of both IHC and RT-QPCR strengthening our interpretation of cancer behavior especially in this new field of lncRNAs.

## 4. Materials and Methods

Breast cancer TMA # BR243w was purchased from US Biomax Inc. (Rockville, MD, USA). A colorectal cancer cell line SW480 xenograft (FFPE) was sectioned using Microtome (Leica Biosystems, Buffalo Grove, IL, USA). FFPE HeLa slides were procured from ACD (#310045, Newark, CA, USA) to use as positive and negative controls.

### 4.1. Research Involving Human Tissues

Human colorectal cancer tissues (FFPE) and pancreatic cancer tissue (FFPE) sections were procured from the Department of Pathology, University of Tennessee Health Science Center (UTHSC, Memphis, TN, USA) as per UTHSC IRB guidelines. Archived FFPE tissues were used in this study. The tissue samples were coded to de-identify any patient information. Therefore, it is considered that “no human subjects” were involved in this study. The study was conducted in accordance with the Declaration of Helsinki, and the “Human Subject Exempt protocol” was approved by the Ethics Committee of the UTHSC, Memphis, TN, USA; project identification code 13-02690-XM, approved on 28 August 2013.

### 4.2. Chromogenic Staining in FFPE Tissues

Formalin fixed (10% neutral buffered formalin, 16–32 h) and alcohol-treated (70% alcohol, 24–48 h) paraffin embedded tissues, sectioned (5–7 μM) and mounted on Superfrost slides (Fisher Scientific, Pittsburgh, PA, USA) were used for lncRNA Z-probe staining. An RNAScope 2.5 HD detection kit (RED) (#322360, ACD, Newark, CA, USA) was used as per manufacturer suggestions with modifications. After baking FFPE slides on a side warmer for 1 h at 60 °C, they were deparaffinized in fresh xylene twice for 5 min, dehydrated in 100% alcohol twice for 1 min and treated with hydrogen peroxide (#322330, ACD) for 10 min at room temperature. Maxed epitopes were retrieved by incubating slides in boiling target retrieval buffer (#322000, ACD) for 25 min (this is a critical step as different tissues require different retrieval times), followed by protease plus (#322340, ACD) treatment for 30 min in the HybEZ (#240200, ACD) incubator preset at 40 °C. Z probes (HsNRON #508481, HsUCA1 #417521, HsMALAT1 #400811) were warmed at 40 °C for 10 min, and amplifiers (#322360, ACD) were normalized to room temperature before the hybridization steps. After aspirating the protease plus, probes were added to the respective tissues and incubated at 40 °C for 2 h in an HybEZ incubator. Hybridized probes bound to complementary bases were amplified through sequential amplification steps from amp-1 to amp-6 followed by incubation at 40 °C in the incubator with washing (#310091, ACD) between each of the amplification steps. Amplifiers tagged with chromogenic labeled dye probes (mix of RED-B and RED-A at 60:1 ratio) after 10 min incubation gave a red signal. Slides were counterstained with 50% hematoxylin for 2 min at room temperature and washed with 0.02% ammonia water followed by distilled water. Slides were finally dried at 60 °C for 15 min on a slide warmer, xylene dipped and mounted with Ecomount (#EM897L Biocare Medical, Pacheco, CA, USA). FFPE mammalian cells sectioned on slides can also be stained using the same method.

### 4.3. Quantitation (ImageJ) Analysis

The stained slides were scanned on a Pannoramic 250 Flash III device (3DHistech, Budapest, Hungary). Images were visualized using CaseViewer 2.2 software (3DHistech, Budapest, Hungary). Four different fields (ROI: Region of interest) of the 10× image were analyzed for the total intensity of the red stain, using ImageJ (nih.gov), representing the corresponding lncRNA staining. The color threshold was adjusted to identify only the red color.

#### Summary of ImageJ Analysis

Each stained tissue image was sectioned into 4 groups and individually analyzed as a region of interest (ROI 1–4). Each sectioned image was then analyzed by measuring the color intensity of individual cells containing multiple punctate dots. The color intensity of each cell in each section was collectively put together by ImageJ software to give an area value of intensity. Each area value corresponds to each ROI per image. Together, all 4 ROIs were then averaged to give an average area of intensity per image.

### 4.4. Statistical Analysis

Statistical analyses were performed using GraphPad Prism 7 (GraphPad Software, La Jolla, CA USA). *p* < 0.05 was considered significant.
